# Improving the effectiveness of anti-aging modalities by using the constrained disorder principle-based management algorithms

**DOI:** 10.3389/fragi.2022.1044038

**Published:** 2022-12-14

**Authors:** Noa Hurvitz, Narmine Elkhateeb, Tal Sigawi, Lilah Rinsky-Halivni, Yaron Ilan

**Affiliations:** ^1^ Faculty of Medicine, Hebrew University and Department of Medicine, Hadassah Medical Center, Jerusalem, Israel; ^2^ Braun School of Public Health, Hebrew University of Jerusalem, Jerusalem, Israel; ^3^ Department of Global Health and Population, Harvard T.H. Chan School of Public Health, Boston, MA, United States

**Keywords:** aging, disorder, artificial intelligence, variability, defective engineering, complex systems

## Abstract

Aging is a complex biological process with multifactorial nature underlined by genetic, environmental, and social factors. In the present paper, we review several mechanisms of aging and the pre-clinically and clinically studied anti-aging therapies. Variability characterizes biological processes from the genome to cellular organelles, biochemical processes, and whole organs’ function. Aging is associated with alterations in the degrees of variability and complexity of systems. The constrained disorder principle defines living organisms based on their inherent disorder within arbitrary boundaries and defines aging as having a lower variability or moving outside the boundaries of variability. We focus on associations between variability and hallmarks of aging and discuss the roles of disorder and variability of systems in the pathogenesis of aging. The paper presents the concept of implementing the constrained disease principle-based second-generation artificial intelligence systems for improving anti-aging modalities. The platform uses constrained noise to enhance systems’ efficiency and slow the aging process. Described is the potential use of second-generation artificial intelligence systems in patients with chronic disease and its implications for the aged population.

## Introduction

Aging is one of the most complex biological processes due to its multifactorial nature, involving genetic, environmental, and social factors. Human life expectancy continually rises, from 67 years in 2000 to 73.4 years in 2019 (World Health Organization, 2022). Sixty years and older subjects account for 25% of the total population in several European countries. By 2050, a quarter of the world population, not including Africa, may reach that proportion (Xu et al., 2019). As life expectancy continues to rise, there is a need to maintain quality of life and a healthy life expectancy (Kennedy et al., 2014).

Keeping the aged generation active and independent reduces the aging dependency rate (Kingston et al., 2017; Motamed-Jahromi and Kaveh, 2020). It is also associated with reducing costs for the senior population suffering from more chronic morbidity and improved quality of life (Foster and Walker, 2015; Maresova et al., 2019).

Variability characterizes the proper function of biological systems (Ilan, 2019a). The constrained disorder principle defines living organisms and non-living systems based on their disorder within arbitrary boundaries (Longo et al., 2012; Ilan, 2020a).

In the present paper, we review several mechanisms of aging and some anti-aging therapies. We reviewed studies that describe the associations between variability and aging by searching for variability and the hallmarks of aging. Studies were selected from the last 10 years using variability and hallmarks of aging search words.

The paper presents the concept of implementing the constrained disorder principle-based second-generation artificial intelligence (AI) systems for improving anti-aging interventions. The paper describes studies that present the concept of using variability for improving systems functions in biology and discuss the applicability of the concept to aging.

## The constrained disorder principle defines living organisms and non-living systems

The combination of order and disorder underlies the normal function of biological systems at the level of the genome, cellular organism, and whole organs (Ilan, 2019a; Ilan, 2019b; Ilan, 2020b; Ilan, 2021b). The constrained disorder principle defines living organisms as disorders bounded by arbitrary dynamic boundaries (Ilan, 2021b). The constrained disorder principle provides systems with the flexibility and adaptability to function under continuously changing internal and external random environments. Per the principle, aging can be viewed as a malfunction of biological systems. Systems malfunctions are associated with a decrease or increase in the degree of disorder, limiting their ability to cope with changes and decreasing their function (Ilan, 2021b).

## Variability characterizes biological systems

The notion of complexity in biological systems evolves from non-linear dynamics and determines the output of physiologic processes that generate variable fluctuations resembling “chaos.” (Ilan, 2019a; Ilan, 2019b; Ilan, 2020b) Non-linear dynamics enable evaluating biological systems’ complexity using the mathematical concepts of chaos theory and fractals (Nicolini et al., 2012). Variability is a hallmark of systems’ complexity that characterizes biological systems (Ilan, 2019a; Ilan, 2019b; Ilan, 2020b). Rearrangements at the genome level comprise genomic alterations that determine the genome stability and plasticity of the genome (Pellestor et al., 2021). Dynamic variability describes the function of microtubules in all cells (Mitchison and Kirschner, 1984; Ilan, 2019c; Ilan, 2019d; Ilan-Ber and Ilan, 2019; Forkosh et al., 2020). Variability is inherent to the immune system’s response to stimuli and characterizes the process of whole organs manifested in heart rate, breathing, blood pressure, and gait variability (Goldberger, 1996; Costa and Goldberger, 2019; El-Haj et al., 2019; Bencivenga et al., 2022).

## The hallmarks of aging are associated with variability: Inter and intra-subject variabilities impact the process of aging

The hallmarks of aging, including genomic instability, telomere attrition, epigenetic alterations, loss of proteostasis, deregulated nutrient-sensing, mitochondrial dysfunction, cellular senescence, stem cell exhaustion, and altered intercellular communication, are of relevance for developing new interventions for aging (López-Otín et al., 2013). New hallmarks of aging comprise autophagy, microbiome disturbance, altered mechanical properties, splicing dysregulation, and inflammation (Lemoine, 2021; Schmauck-Medina et al., 2022).

Aging is a multifactorial biological process, and the altered complexity and variability contribute and are reflected by the additional aging machineries (Xu et al., 2019). Each mechanism alone does not explain the complexity of aging, and this is a multifactorial process. A progressive function impairment during aging increases vulnerability to environmental challenges and is a growing risk of disease and death (Kirkwood, 2005).

Variability characterizes several of the hallmarks of aging (Martinez-Jimenez et al., 2017; Crimmins, 2020; Guerville et al., 2020).

1. **Variability characterizes genomic instability and transcriptional dynamics:** Genome instability is the tendency of the genome to undergo alterations in DNA information content through mutation and characterizes aging. Single-cell sequencing-based methods provide quantitative assays for estimating the possible functional effects of mutations accumulating during aging in various tissues and organs (Vijg and Suh, 2013). DNA damage accrual triggers cellular senescence and metabolic changes that promote a decline in tissue function and increased susceptibility to age-related diseases (Niedernhofer et al., 2018). Distinct cellular processes result in cell-to-cell variation in gene expression (Mendenhall et al., 2021). These processes range from silencing individual alleles of a gene to differences in a cell’s reception of a signal and even to a cell’s general ability to execute the genetic program by translating genetic information into protein. Cells can perceive signals differently, either due to chance differences in perception or differences in signaling capacity for a particular signaling pathway, which may be stochastic or epigenetic in origin (Mendenhall et al., 2021). A three-tissue single-cell RNA-seq study found that there is both increased and decreased variation in gene expression with age that is both gene and cell type dependent (Kimmel et al., 2019). Aging increased expression heterogeneity across populations of cells, implying that increased cell-to-cell transcriptional variability is a hallmark feature of aging (Bhadra et al., 2020). In young animals, immunological activation drives a conserved transcriptomic switch resulting in tightly regulated gene expression, characterized by a strong up-regulation of a core activation program, coupled with a decrease in cell-to-cell variability (Martinez-Jimenez et al., 2017).
2. **Variability in telomere attrition:** Telomeres are the terminal nucleoprotein complexes of chromosomes that protect against degeneration, reconstruction, fusion, and loss and contribute to the pairing of homologous chromosomes (Blackburn, 2005; Xu et al., 2019). The telomeres get shorter as cells divide and more profoundly in the presence of oxidative stress. Telomerase is an enzyme responsible for maintaining the length of telomeres. Telomerase-deficient mice are susceptible to progressive tissue atrophy, stem-cell depletion, organ system failure, and impaired tissue injury responses, which are reversed by telomerase reactivation, suggesting that telomere shortening is a primary determinant of aging (von Zglinicki, 2002). TERT, the gene coding for the enzyme telomerase, is a telomere-associated protein involved in the Wnt pathway. The telomerase protein regulates the Wnt/β-catenin signaling directly, and TERT acts as a co-factor in the β-catenin complex (Zhou et al., 2014). Expression of hTERT contributes to slowing down telomere shortening (Zheng et al., 2014; Kim et al., 2016).

Telomere attrition indicates accelerated biological aging (Maxwell et al., 2011). Telomere attrition contributes to increased morbidity and mortality in subjects with chronic and aging-related diseases (Xu et al., 2019; Wang et al., 2018a; D'Mello et al., 2015; Dai et al., 2014). Mitochondrial dysfunction and reactive oxygen species are associated with telomere attrition (Liu et al., 2002). Reintroducing telomerase in mice models reverses the tissue degeneration and the accelerated-aging phenotype (Jaskelioff et al., 2011; Sahin et al., 2011).

Shortening telomerase is associated with disturbances in the immune system and inflammation, increased pro-inflammatory cytokine tumor necrosis factor α that characterizes older adults, and increased risk for Alzheimer’s disease, diabetes, and cardiovascular diseases (von Zglinicki, 2002; Fulop et al., 2017; Herrmann et al., 2018; Spittau, 2017). Telomerase lengthening occurs with low alcohol consumption, cigarette smoking, a healthy diet, and physical activity (Latifovic et al., 2016; Sellami et al., 2021).

Variability in telomere length was described between species, individuals within a species, and even among different tissues in a certain individual (Foote et al., 2013). A newborn’s telomere length is considerably variable and is influenced by environmental and genetic factors such as vitamin B12 levels, active smoking during pregnancy, elevated fasting blood glucose, and maternal telomere length (Chen et al., 2022). Although studies have shown a correlation between telomere shortening and aging, the shortening rate varies across the different tissues in the same individual. This variation in relative telomere length is attributed to cell composition, measurement error, lifestyle, and environmental factors with variable effects across tissues (Demanelis et al., 2020). There is a balance between hTERT expression that prevents telomere shortening and the onset of cancer that might be triggered by hTERT overexpression (Cristofari and Lingner, 2006; Bernardes de Jesus and Blasco, 2013). It is an example of the difficulty of correcting a mechanism without impacting the biological equilibrium and triggering harmful side effects.

3. **Variability in epigenetic alterations:** DNA methylation, histone modifications, and noncoding RNA species affect the vast majority of nuclear processes, including gene transcription and silencing, DNA replication and repair, cell cycle progression, and telomere and centromere structure and function (Gonzalo, 2010). Alterations of these epigenetic mechanisms represent a significant factor in the pathophysiology of aging (Vijg and Suh, 2013; Martinez-Jimenez et al., 2017; Niedernhofer et al., 2018). Specific epigenetic signatures can be biomarkers for age-associated disorders and therapeutic targets (Galow and Peleg, 2022).
4. **Variability in loss of proteostasis:** Accumulation of intracellular damage is a hallmark of aging. Maintenance of protein homeostasis, or proteostasis, is attained through precisely coordinated systems that rapidly correct unwanted proteomic changes. Malfunctions of the proteostasis networks responsible for coordinated protein homeostasis intracellularly, in between cells and across organs, affect age-associated diseases (Kaushik and Cuervo, 2015). Young cells are kept healthy by protein synthesis, degradation, and chaperoning. The capacity of cells to maintain proteostasis undergoes a decline during aging (Klaips et al., 2017). Aging is associated with negative processes of oxidative damage accumulating randomly in the cell’s proteins, healthy synthesis, and degradation slow down, and chaperone capacity is exceeded (Santra et al., 2019). The chaperones are distracted trying to fold irreversibly damaged proteins, accumulating misfolded and aggregated proteins in the cell (Santra et al., 2019).
5. **Variability in deregulated nutrient-sensing:** Nutrient sensing pathways become deregulated with age and are a link between diet and aging. MicroRNAs are regulators of cellular functions, target genes encoding proteins and enzymes belonging to nutrient sensing, and can be modified by diet (Mico et al., 2017). Nutrient sensing contributes to pathways in cognitive aging (de Lucia et al., 2020). Modifying nutrient-sensing pathway therapeutics, particularly growth hormone secretagogues, can improve cognitive outcomes (Kioussis et al., 2021).
6. **Variability in mitochondrial dysfunction and reactive oxygen species:** Mitochondria are essential players in the aging process, and most aging-related diseases, particularly neurodegenerative diseases, have mitochondrial involvement. Mitochondria are involved in energy production through oxidative phosphorylation and play a role in intracellular homeostasis, calcium balance, and the metabolism and interconversion of our dietary substrates, fats, proteins, and carbohydrates, in the fed and fasting states, which are relevant for aging (Haas, 2019). Mitochondrial genome alterations are reported to be associated with numerous disorders affecting nearly all tissues (Volobueva et al., 2018). Reactive oxygen species (ROS) are produced during normal aerobic metabolism and accumulate with age leading to oxidative damage of genomic DNA, proteins, and cellular components (Harman, 1956). A correlation between somatic mutations and other DNA damage and ROS may play a role in aging. A strong association between longevity and efficient DNA repair mechanisms is described (Promislow, 1994). A higher level of PARP-1, a key component in the cellular response to DNA damage, was associated with a prolonged life span (Grube et al., 1992). Increased ROS production, mitochondrial DNA (mtDNA) damage accumulation, and progressive respiratory chain dysfunction may underlie the aging procesess (Dufour and Larsson, 2004). A gradual accumulation of mitochondrial DNA damage during aging increase the production of ROS, leading to a decline in energy production and damage to proliferating tissues due to the vulnerability of DNA molecules during replication (Kirkwood, 2005). A study of the variability of oxidative stress biomarkers in healthy individuals’ urine found intra-individual variability of 29%–149% and inter-individual variability of 112%–272% (Martinez-Moral and Kannan, 2019).
7. **Variability in cellular senescence:** Senescence occurs in response to danger signals, activated oncogenes, and metabolic abnormalities (Schafer et al., 2017). Senescence-associated secretory phenotype (SASP) implies that a senescent cell produces and secretes cytokines to affect its microenvironment (Siddiqui et al., 2021). Statins, Metformin, Rapamycin, and JAK1/2 inhibitors act as SASP inhibitors, improving an aging state and alleviating age-related diseases in mammals at different level (Ayad et al., 2018; Ala and Ala, 2021; Franzin et al., 2021). Extracellular vesicles (EVs) are small membranous vesicles released into the extracellular environment by most cells. EVs are participants of a SASP. Senescent cells secrete more EVs when considering the cell type and stimulation (Misawa et al., 2020; Wallis et al., 2020). Cell senescence is influenced by ROS damage, error propagation and catastrophe, loss of cell proliferative capacity, telomere shortening, chromosomal damage, and non-enzymatic glycation of macromolecules. The numerous sources of damage lead to variable patterns of senescence within and between different organs within individuals, individuals within a species, populations within species, and between species (Crews and Ice, 2012). Analysis of individual cells identifies cell-to-cell variability following induction of cellular senescence (Wiley et al., 2017). The cell-to-cell variability resulted in a loss of correlation among the expression of several senescence-associated genes.
8. **Variability in stem cell exhaustion:** Ageing may be caused, in part, by the depletion of stem and progenitor cells that govern tissue renewal. The potential causes of stem and progenitor cell attrition are numerous, and these cells are lost due to naturally occurring DNA damage and the obligate checkpoint responses that follow. Failure to launch adequate responses to DNA damage is associated with aging processes (Ruzankina and Brown, 2007). Manipulation of exhausting adult stem cells is being evaluated to treat aging (Boyette and Tuan, 2014).
9. Cellular aging leads to functional heterogeneity of hematopoietic stem cells (Glauche et al., 2011). The altered composition of inflammatory signals from immune dysfunction in aging prevents regeneration limiting stem cell function (Oh et al., 2014). Among stem cells of highly proliferative tissues such as bone marrow and gut epithelium, damaged cells pose a significant threat to the organism’s health because of their potential to give rise to large numbers of mutant, potentially malignant daughter cells. In aged mice, stem cells that exhibit functional deterioration show increased levels of apoptosis in response to low-dose genotoxic stress (Herndon et al., 2002).
10. **Variability in intercellular communication:** Intercellular communication is altered in aged organs and can lead to some diseases and disabilities of aging (Fafián-Labora and O’Loghlen, 2020). Ventricular intercellular coupling transverse to the fiber axis in aged hearts is reduced (Dhein and Hammerath, 2001). Altered cell-cell communication contributes to the development of fibrosis in aging (Thannickal, 2013).
11. **Variability in autophagy:** Autophagy promotes the degradation of damaged proteins and organelles in cells and prevents the accumulation of harmful substances. Autophagy is activated under stressful conditions and attenuates age-related organismal damage and functional decline in aged mice (Koutouroushis and Sarkar, 2021; Li et al., 2021). The variability of autophagy is associated with cell death susceptibility (Loos et al., 2013). The cell’s capacity for autophagic degradation declines with age (Gelino and Hansen, 2012). Regulation of autophagy is an anti-aging strategy (Bao et al., 2020). The loss of function of proteosomes and chaperones results in the accumulation of damaged proteins contributing to various age-related disorders, including cataracts, Alzheimer’s, and Parkinson’s diseases (Carrard et al., 2002; Soti and Csermely 2003). In aging, cellular repair mechanisms gradually fail over time, accumulating molecular and cellular damage and loss of function.
12. **Variability in microbiome:** An imbalance in microbiome diversity leads to inflammatory responses to the gut mucosa during aging, leading to chronic inflammation (Bulut et al., 2021; Du et al., 2021; Zhou et al., 2021). Variability in microbiome disturbance is characterized by altered diversity of the microbiota (Ilan, 2019e). Aging is associated with decreased microbiota diversity, increased tryptophan metabolism contributing to long-term stimulation of the immune system, and decreased immune-system function, termed immunosenescence (Wu et al., 2021). A reduction in the relative abundance of Bifidobacteria and an increasing abundance of Bacteroides and Enterobacteriaceae characterize aging. Long-term Lactobacillus and Bifidobacterium dietary supplementation enhance memory and change the brain’s metabolism in aged rat (O'Hagan et al., 2017). Increased Eubacterium limosum is associated with longevity. Tryptophan is a microflora metabolite and regulates the balance between gut immune tolerance and gut microbiota maintenance, and its levels decrease in older adults (Musumeci et al., 2017; van Beek et al., 2017; Gao et al., 2018). Augmenting tryptophan metabolism correlates with aging, and older people show lower serum tryptophan level (Collino et al., 2013).
13. **Variability in mechanical properties:** During aging, changes occur in the collagen network leading to pathological phenotypes in the skeletal, vascular, and pulmonary systems (Panwar et al., 2015). Modifying collagen compromises its biomechanical integrity and affects the degradation of bone and tissue and extracellular matrix aging.
14. **Variability in splicing dysregulation:** Age-associated splicing changes across tissues are linked with age-associated diseases. RNA splicing and its regulators play a role in biological aging and diseases associated with aging or aging-like phenotype (Angarola and Anczuków, 2021). In a study of 9,869 tissue-specific age-associated splicing events of 7 distinct types, genome-wide splicing profiles better predicted biological age than the gene and transcript expression profiles. Age-associated splicing predicted the relative ages of cells (Wang et al., 2018b).
15. **Variability in inflammation:** Inflamm-aging refers to low-grade chronic inflammation characterized by an increased level of cytokines and pro-inflammatory markers accompanying the aging process and resulting from continuous inflammatory activation signaling in the presence of damaged organelles that accumulate in old organisms (Franceschi et al., 2007; Franceschi et al., 2017a; Conte et al., 2020). The long-term stimulation of the immune system leads to a decline in immune-system functioning and immunosenescence (Vaiserman et al., 2017). High levels of IL-6 and TNF-α were seen in aged skin, heart, brain, and kidney, and were associated with the impaired immune system, accumulation of senescent cells, different epigenetic modifications, and gut microbiota dysbiosis. (Williams et al., 2009; Coppe et al., 2010) Telomers shortening, gut dysbiosis, oxidative stress, decreased T cell function, thymus involution, and hormonal changes contribute to inflamm-aging (Guigoz et al., 2008; Santoro et al., 2020). The self-debris from unhealthy or dead cells are produced at a higher rate in aged tissues, accumulating senescent cells and a pro-inflammatory secretome (Coppe et al., 2010; van Deursen, 2014). Inflamm-aging is associated with atherosclerosis, dementia, diabetes mellitus, cardiovascular changes, Alzheimer’s disease, Parkinson’s disease, depression, osteoarthritis, sarcopenia, and cancer (Castelo-Branco and Soveral, 2014; Franceschi et al., 2017b; Franceschi et al., 2018a; Franceschi et al., 2018b; Teodori et al., 2019; Franceschi et al., 2020). Variability characterizes many of these processes (Lemoine, 2021; Schmauck-Medina et al., 2022). In mice’s immune system, aging was associated with increased cell-to-cell variability in gene expression. (Martinez-Jimenez et al., 2017) Analysis of epigenetic alterations of single immune cells in humans has shown that aging is associated with increased variability in chromatin modification profiles of different cells within an individual (Cheung et al., 2018). Hence, the immune system appears to gain complexity and increased variability with aging.
16. **Variability in neuro-cardiovascular regulatory mechanisms**: Using fractal methods on data extracted from wearable devices to quantify the variability in fluctuating daily physical activity patterns has shown that aging and mortality were associated with decreased variability (Raichlen et al., 2019; Bencivenga et al., 2022; Lee et al., 2022). Physiological complexity was proposed as a biomarker of aging, and interventions that aim to regain complexity in physical activity might slow the aging process. (Raichlen et al., 2019) Heart rate variability (HRV) is the beat-to-beat variation in successive RR intervals on an electrocardiographic (ECG) recording. HRV reflects the autonomic nervous system’s heart rate regulation and comprises an inherent cardiac variability (Goldberger, 1996; Costa and Goldberger, 2019). Non-linear dynamics uses HRV-based parameters for the prognostic significance of HRV in older adults (Nicolini et al., 2012). The chaos theory characterizes the effects of aging on cardiac dynamics by determining the HRV based on fractal scaling and non-linear dynamics. The age-related changes are similar to fluctuations in measures based on fractal scaling and complexity chaos theory. Healthy aging is associated with higher regularity and altered fractal scaling consistent with a loss of complex variability (Pikkujamsa et al., 1999). Non-linear and linear measures of HRV are altered with aging in supine and standing postures. Increasing age led to a reduction in sample entropy in supine posture. Standing compared to supine posture had a more negligible effect on healthy aging (Vuksanovic and Gal, 2005).

In contrast, other studies showed increased complexity and variability during aging (Kyriazis, 2003). Recent studies showed that HRV indices do not reflect the cardiac neuroautonomic function due to the assumption that the amplitude of variations in sinus normal-to-normal intervals are associated with the degree of vagal tone modulation. The vagal activity decreases with aging, reflected by a reduction in short-term HRV values. However, the data showed a counterintuitive increase in short-term/high-frequency variability in high-risk individuals (Huikuri and Stein, 2012; Drawz et al., 2013; Raman et al., 2017). The high HRV in diminished vagal tone modulation settings was termed the “HRV paradox.” (Costa et al., 2017a; Hayano et al., 2019; Hayano and Yuda, 2019; Yuda et al., 2020a; Yuda et al., 2020b; Yuda et al., 2021) Heart rate fragmentation (HRF) is a property of HR dynamics reflecting the altered sinoatrial pacemaker-neuroautonomic function that resolves this paradox (Costa et al., 2017a; Costa et al., 2017b). In older adults, HRF manifests itself by an increase in the density of heart rate acceleration sign changes and is not explainable by modulation of the cardiac vagal tone (Costa et al., 2017a; Costa et al., 2017b; Costa et al., 2018; Costa and Goldberger, 2019; Costa et al., 2021; Omoto et al., 2021). HRF supports an underlying variability that characterizes cardiac function, increases in older adults, and correlates with cardiovascular events (Costa and Goldberger, 2019). Interventions that slight or reverse aging decrease HRF (Gorelick et al., 2011; Collins et al., 2012; Alonso et al., 2013; Costa et al., 2018; Costa and Goldberger, 2019; Costa et al., 2021).

Blood Pressure Variability (BPV) is associated with organ damage and exerts independent predictive value on cardiovascular events, neurocognitive impairment, metabolic disorders, sarcopenia, and frailty (Bencivenga et al., 2022). BPV is associated with several age-related mechanisms, including genomic instability, epigenetic modification, and mitochondrial oxidative damage (Woo et al., 2018; Ma et al., 2020; Bencivenga et al., 2022; Jia et al., 2022). Altered BPV is associated with arterial stiffness and autonomic dysfunction in older adults. Subclinical inflammation associated with aging is also related to BP dysregulation *via* altered endothelial function (Seals et al., 2011; Donato et al., 2018).

Neural activity signals recorded by resting EEG and fMRI have shown an overall trend of increased entropy and variability with aging, reflecting a loss of synchronization of the neuronal interconnections (Sleimen-Malkoun et al., 2014; Wang, 2020; Alù et al., 2021). Interventions that aim to reduce the entropy of EEG signals were hypothesized to direct personalized rehabilitation programs for older persons. (Alù et al., 2021) However, several task-related EEG patterns have demonstrated reduced variability with aging, and fMRI scans of patients with advanced neurodegenerative diseases have also shown decreased entropy compared to healthy individuals^157 158^.

17. **Alterations in longevity proteins:** Numerous pathways and proteins are involved in the aging process (Niedernhofer and Robbins, 2008). The transcription of the NF-κB path is involved in cellular, development, and inflammatory pathways and underlies age-related diseases. It is upregulated in neurodegeneration, atherosclerosis, osteoarthritis, and cardiovascular dieases (Hayden and Ghosh, 2008; Kounatidis et al., 2017). The Wnt pathway is involved in the developmental cell processes, such as cell polarity, cell-fate determination, and progenitor cell proliferation (Ring et al., 2014). Adiponectin is an adipokine with anti-aging effects. Adiponectin-activated pathway contributes to obesity, diabetes, heart disease, and Alzheimer’s and reduces life span (Iwabu et al., 2015; Kim et al., 2020). Klotho is an aging suppressor gene, and its knockout in mice leads to accelerated aging and sarcopenia (Xiao et al., 2004; Laszczyk et al., 2017). Sirtuins are proteins in cell cycle regulation, DNA damage repair, inflammation response, mitochondrial functions, genomic stability, longevity, inflammation alleviation, and metabolic homeostasis (Fang et al., 2019; Watroba and Szukiewicz, 2021). SIRT1 and SIRT6 are longevity regulators, and caloric restriction alleviates aging-related changes and increases their expression (Kanfi et al., 2008). SIRT1 attenuates age-related neurodegenerative diseases and glucose resistance in mice (Alcendor et al., 2007). SIRT1 augments DNA methylation which is, which is helpful for longevity (Watroba and Szukiewicz, 2021). SIRT6 knockout leads to premature aging and death, and its overexpression extends lifespan (Mostoslavsky et al., 2006; Kanfi et al., 2012). Variation in SIRT3 variation in males affects human longevity (Rose et al., 2003).

The growth hormone induces the insulin/insulin-like growth factor (IGF) pathway. It controls tumorigenesis, diabetes, and cardiovascular diseases associated with aging (Tullet et al., 2008; Lee and Kim, 2018). Growth hormone deficiency or resistance extends lifespan in mice, with a decreased IGF-1 level (Bartke, 2008). Inhibition of IGF-1/insulin signaling extends the lifespan of *C. elegans* (Bartke, 2008; Tullet et al., 2008).

The mammalian target of rapamycin (mTOR) kinase mTOR is a serine/threonine protein kinase. mTOR is a regulator of glucose homeostasis, lipid homeostasis, immune function, brain function, cancer, and a senescence-associated secretory phenotype (SASP), associated with longevity and aging (Papadopoli et al., 2019). The mTOR pathway and its two principal components (MTORC1, MTORC2) act as interconnected sensors that regulate metabolism, cellular growth, apoptosis, autophagy, and inflammation (Laplante and Sabatini, 2012). mTOR-dependent pathways control critical biological processes based on upstream signals from signaling cascades triggered by numerous receptors, including FGF, EGF, IGF, insulin, and TGF-beta (Laplante and Sabatini, 2012; Saxton and Sabatini, 2017; Conciatori et al., 2018). An inhibitor of mTOR, rapamycin, extends the lifespan of mice (Shimobayashi and Hall, 2014). It emphasizes the systemic nature of the cellular environment, which makes the design of anti-aging therapies challenging.

18**. Alterations of the metabolic pathways in aging:** Aging is linked with energy intake and expenditure, body composition, physical activity, and controlling energy balance (Roberts and Rosenberg, 2006). Nicotinamide adenine dinucleotide (NAD+) is a cellular factor participating in metabolic pathways, and its levels are reduced with aging. Calorie restriction (CR) increases NAD + levels, increasing lifespan. An NAD + precursor, nicotinamide riboside, or nicotinamide mononucleotide dietary supplementation increase longevity in mice (Abdellatif et al., 2021).

Overall the data supports the importance of altered variability in biological systems in the aging process. It supports the notion that intra and inter-subject variabilities are relevant to the aging hallmarks and processes. It goes along with the constrained disorder principle, which means that aging is associated with out-of-borders disorders. In some cases, it can involve a decrease and, in others, an increase in variability.

## Heterogeneity of aging

Aging involves different degrees of inter-individual heterogeneity and is not a homogeneous process. Inter-individual heterogeneity refers to the differences between subjects in the aged group and between old and younger individuals. In addition to the inter-individual variability, alterations in the degree of intra-subject variability underlie the aging process. Age-associated increase in inter and intra-individual heterogeneity is substantiated by health-related quality of life, independence in activities of daily living (ADLs), psychomotor and cognitive tasks, and gene expression in the human brain. (Shammi et al., 1998; Lowsky et al., 2014; Kedlian et al., 2019) Older adults are more heterogeneous than younger adults (Nguyen et al., 2021). A large-scale study examined the heterogeneity of 34 health characteristics in eight domains amongst age groups ranging from 45 to 86 years. Systolic blood pressure, bone mineral density, some physical performance measures, chronic condition count, and most of the laboratory values tested were among the variables that demonstrated a statistically significant increase in heterogeneity with age. Several characteristics demonstrated a decrease in heterogeneity, and some showed no association of heterogeneity with age (Nguyen et al., 2021).

Several environmental factors contribute to the heterogeneity of aging, including socioeconomic status, social engagement, exercise, pharmacological treatment, and diet (Lowsky et al., 2014; Tejedor and Fraga, 2017). These factors impact the accumulation of degenerative processes in the aging individual. A higher degree of heterogeneity was demonstrated in the older population in their response to medications (McLachlan et al., 2009). Age-related mechanisms of altered drug metabolism, distribution, and interactions were proposed to underlie this phenomenon (McLachlan et al., 2009).

Chronological age is a poor predictor of adverse health outcomes and does not represent the complexity and variability of aging (Vaillant and Mukamal, 2001; Lowsky et al., 2014; Khan et al., 2017; Mitnitski et al., 2017). When examining genetically identical living models (single cells, tissues, or organisms), heterogeneity in aging is prominent, even under controlled environmental conditions, suggesting that additional factors contribute to this phenomenon (Kedlian et al., 2019; Neupert and Zhu, 2020; Nguyen et al., 2021; Shadel et al., 2021). Aging is associated with a decline in function, yet many older adults remain functional with no disability even at a very advanced age (Vaillant and Mukamal, 2001; Lowsky et al., 2014).

Potential biomarkers for aging include physical function measures, a variety of standard laboratory tests reflecting cardiovascular, hormonal, or inflammatory status, and markers based on DNA/epigenetic alterations, protein modifications, and miRNAs (Bürkle et al., 2015; Wagner et al., 2016; Tejedor and Fraga, 2017). Frailty explains the heterogeneity in life expectancy in individuals of the same age. It is a phenotype predictive of adverse health outcomes in older people. (Zaslavsky et al., 2013; Ribeiro et al., 2020) The “frailty index” evolved from the understanding that heterogeneity in health is measured using a deficit accumulation approach. It quantifies biological age by counting a range of clinically apparent health deficits, dividing the result by the total number of items measured (Mitnitski et al., 2017). The “complexity theory” may explain the dynamic nature of frailty (Lipsitz and Goldberger, 1992; Zaslavsky et al., 2013). It suggests that a decline in the “complex network of communicating physiological signals” lowers the ability to cope with stressors and leads to greater clinical susceptibility or frailty.

Overall the data support the relevance of inter and intra-subject variability in the pathogenesis of aging.

## The constrained disorder principle defines aging as moving outside the boundaries of variability

Aging is associated with altered variability in biological systems (Gusso et al., 2019). Per the constrained disorder principle, a marked increase or decrease in the degree of variability, or getting outside of the constrained borders, underlie the aging process.

A loss of physiological complexity from molecular to cellular and tissue to organismic levels was proposed to be associated with aging, leading to an impaired ability to adapt to stress and numerous internal and external stimuli (Kyriazis, 2003). Age-related loss of complex variability is reflected by multiple processes, including cardiovascular control, electroencephalographic potentials, and pulsatile hormone release (Kyriazis, 1991; Lipsitz and Goldberger, 1992). The non-linear prediction method combined with Gaussian-scaled surrogate data can determine the chaotic behavior of the interspike interval of ventral tegmental area dopaminergic neurons in rats as a function of age. The complexity of action potentials and the chaotic content of ventral tegmental area dopamine neurons are reduced with aging (Di Mascio et al., 1999).

The correlation between aging and the variability or entropy of different cardiovascular parameters, including systolic and diastolic blood pressure, heart rate, and oxygenated hemoglobin, was examined in several studies. Some of these have demonstrated reduced variability with aging or frailty, while others have shown the reverse association (Fukusaki et al., 2000; Viola et al., 2011; Nemati et al., 2013; Toosizadeh et al., 2022; Ogliari et al., 2016; Knight et al., 2020; Lee et al., 2022).

## Anti-aging interventions: Pre-clinical studies are inconsistent

Numerous anti-aging interventions were effective in pre-clinical studies, while only a minority showed some effect in controlled clinical studies (Blagosklonny, 2017; Klimova et al., 2018; Piskovatska et al., 2019).

Calorie restriction (CR) without malnutrition is one of the most studied anti-aging interventions (Bao et al., 2020). Caloric restriction (CR) refers to the total amount of calories that limit daily intake when providing sufficient nutrients such as essential amino acids and vitamins to ensure the organism does not suffer from malnutrition (Bao et al., 2020). Fasting and CR activate autophagy and upregulate autophagy markers improving organ function (Bi et al., 2018). CR extends the life span in several models, including mice, yeasts, flies, and worms. (Gomez-Delgado et al., 2018) The mechanism involves activating AMP protein kinase (AMPK) and sirtuins, inhibiting IGF-1 signaling, and inhibiting the mammalian target of rapamycin (mTOR). (Son et al., 2019) CR in monkeys prevented hyperinsulinemia and improved longevity by mitigating age-related diseases (Bodkin et al., 2003). CR alleviated morbidity and mortality among monkeys with reduced incidence of diabetes, cancer, cardiovascular disease, and brain atrophy and improved age-related and all-cause survival. (Colman et al., 2009; Colman et al., 2014) CR prevents the aggregation of degenerative mitochondria in aging cells by activating autophagy (Bao et al., 2020).

“CR mimetics” (CRM) are compounds that activate signaling pathways and enhance longevity, similar to CR. The glycolytic inhibitor 2-deoxy-D-glucose (2DG) is a CRM that extends lifespan in rats and *C. elegans* and exerts benefits in cerebral ischemic damage and neurodegenerative disorders (Saraswat and Rizvi, 2017).

Rapamycin, a macrolide isolated from *Streptomyces* bacteria, is CRM used as an immunosuppressive agent. It acts as an mTOR inhibitor that modulates autophagy, delays aging and increases lifespan in fruit flies and mice (Harrison et al., 2009; Bjedov et al., 2010).

Polyphenols, curcumin, and Resveratrol are CRM molecules. Curcumin is a food additive known for its anti-aging properties. It inhibits lipid peroxidation, neutralizes several oxidative molecules, including ROS, increases HO-1 in the brain and vascular cells, and may inhibit NFkB activation (Calabrese et al., 2012). Curcumin increases lifespan in *D. melanogaster* (Brouet and Ohshima, 1995; Suckow and Suckow, 2006; Lee et al., 2010a; Lee et al., 2010b; Lee et al., 2010c; Shen et al., 2013).

Resveratrol activates Sirt1 and is an anti-inflammatory, anti-tumoral, and anti-oxidant agent (Gomez-Delgado et al., 2018). It extends the lifespan in *Drosophila* when fed a high-fat diet related to NAD + dependent histone deacetylase, Sir2 (Bass et al., 2007; Wang et al., 2013). Low resveratrol doses improved vascular function and aerobic capacity and decreased senescence markers in rats but did not extend lifespan (da Luz et al., 2012). It benefits DM2, cardiovascular morbidity, Alzheimer’s disease, and cancer (Son et al., 2019).

The IGF-1 signaling pathway has importance on longevity, and a reduced GH/IGF-1 axis protects from cancer, diabetes, and age-related diseases. GH receptor antagonist (pegvisomant) reduces IGF-1 circulating levels, increases insulin sensitivity, and impacts longevity (Trainer et al., 2000; Junnila et al., 2013).

Metformin is a biguanide that reduces gastrointestinal glucose absorption, inhibits gluconeogenesis, is an antioxidant, and acts as CRM (Glossmann and Lutz, 2019; Mohammed et al., 2021). It increases the activity of antioxidant enzymes in erythrocytes and malondialdehyde reduction, decreasing oxidative stress in T2D subjects (Bang et al., 2014; Abad-Jimenez et al., 2020; Ahmed Mobasher et al., 2020). It reduces inflammation, activates autophagy, diminishes oxidative damage, and acts as an anti-aging agent (Gomez-Delgado et al., 2018; Son et al., 2019). Metformin reduces the amounts of advanced glycation end-products (AGEs) that contribute to cellular senescence and target-organ damage in T2D, neurodegeneration, and inflammation (Haddad et al., 2016; Diamanti-Kandarakis et al., 2017).

Aspirin acts as a CRM. Besides its known anti-inflammatory activity, it activates AMPK and reduces mTORC1, enhancing autophagy (Williams et al., 2009; Saraswat and Rizvi, 2017). It is beneficial in extending the median lifespan in mice (Strong et al., 2008; Madeo et al., 2019). Non-steroidal anti-inflammatory drugs extended the lifespan in various model organisms increasing the locomotor activity and stress resistance in *Drosophila* (Poole et al., 2004; Danilov et al., 2015). The cyclooxygenase-2 (COX-2) inhibitor celecoxib extends *C. elegans* lifespan (Ching et al., 2011). Celecoxib is a histone deacetylase (HDAC) inhibitors (Pasyukova and Vaiserman, 2017).

Plant-based diets modulate energy-sensing pathways and involve AMPK, target mTOR, nuclear factor erythroid 2-related factor 2 (Nrf2), sirtuin-1, fox head box O(FOXO), and peroxisome proliferator-activated receptor-gamma coactivator-1 alpha (PGC-1α) (Martel et al., 2021). Plant-rich diets that include phytochemicals activate anti-oxidant gene expression. Sulforaphane, found in edible plants (i.e., broccoli), induces the classical Nrf2-target gene and enhances neuroprotection. Sulforaphane administration attenuates water channel aquaporin-4 (AQP4) loss in the traumatic brain injury model (Zhao et al., 2005).

Protein restriction affects mTORC1 regulation and circulating branched amino acids. (Solon-Biet et al., 2014) Restriction of several amino acids impacts several metabolic processes, i.e., methionine reduced liver steatosis in mice. The ketogenic diet (KD) increases protein acetylation, regulates mTOR signaling, reduces midlife mortality, and improves memory in aging mice (Newman and Verdin, 2017; Roberts et al., 2018). KD promotes longevity in mice with enhanced motor function (Balietti et al., 2010; Kovacs et al., 2021). A high-fat diet and a KD reverse lifespan shortening and attenuate the premature aging phenotype in a mouse model (Xu et al., 2019).

Vitamin E or α-tocopherol extends the lifespan of flies (Dias-Santagata et al., 2007). Vitamin C, an anti-oxidant, did not affect nematode lifespan (Harrington and Harley, 1988). Anti-oxidant dietary supplementation augments the *Drosophila melanogaster*, longevity (Brack et al., 1997). Riboflavin supplementation and melatonin prevent oxidative damage (Zou et al., 2017). Nordihydroguaiaretic acid (NDGA) extends the lifespan of flies (Miquel et al., 1982). Anti-oxidant intakes, such as polyphenols, can increase the telomere length (Maleki et al., 2020).

Metabolic intermediates include oxaloacetate supplementation, malate, TCA metabolite, fumarate and succinate, and α-ketoglutarate, a tricarboxylic acid cycle intermediate that increases the lifespan of *C. elegans* (Williams et al., 2009).

Selective elimination of senescent cells using senolytic agents is explored as an anti-aging strategy (van Deursen, 2014; Saraswat and Rizvi, 2017; Melo Pereira et al., 2019). Natural compounds (i.e., quercetin, piperlongumine, and fisetin), BCL2 inhibitors, FOXO4-interacting peptides, HSP90 inhibitors, histone deacetylase inhibitors, and Tyrosine Kinase inhibitors show senolytic activity. Dasatinib, a tyrosine kinase inhibitor, combined with quercetin, increased lifespan and improved daily function in mice. (Ogrodnik et al., 2017; Gomez-Delgado et al., 2018; Xu et al., 2018)

Lithium extends *C. elegans* lifespan *via* an epigenetic mechanism by altering the expression of genes encoding nucleosome-associated functions (McColl et al., 2008; Wan et al., 2013). Lithium in low doses but not at high dosages extended fly lifespan by inhibiting glycogen synthase kinase-3 (GSK-3) and activating the nuclear erythroid 2-related factor (NRF-2) transcription factor (Castillo-Quan et al., 2016).

Minocycline, a metabolite in the formation of kynurenine from tryptophan, exerts anti-inflammatory, antioxidant, and neuroprotective properties increasing *Drosophila* survival (Oxenkrug et al., 2012).

Natural product molecules can delay the aging process by influencing metabolic pathways (Muzammil et al., 2021). Blueberry polyphenols, quercetin, and ginkgo *biloba* extracts extend *C. elegans* lifespan (Wilson et al., 2006; Pietsch et al., 2009). Cocoa increased lifespan in *D. melanogaster* and demonstrated anti-oxidant properties (Bahadorani and Hilliker, 2008). Rosemary (*Rosmarinus officinalis L.*) is known for its anti-oxidant activity, causes longevity in *Drosophila*, and is associated with increased superoxide dismutase and catalase activities (Wang et al., 2017). *Ludwigia octovalvis* (LOE) is a rich source of antioxidants, including polyphenol compounds, phytosterols, and squalene, and it extends the lifespan of fruit flies (Lin et al., 2014).

Epigenetic alterations affect senescence and cellular mitotic competence. (Herrera et al., 2008; Melo Pereira et al., 2019) Enhanced histone deacetylases (HDAC) activity is involved divergent processes, including inflammation, differentiation, apoptosis, and autophagy. Inhibitors of HDACs extend lifespan (Chen et al., 2015; Pasyukova and Vaiserman, 2017). A lifespan-extending effect in *Drosophila* was shown for 4-phenylbutyrate treatment by histone (de)acetylation (Kang et al., 2002). Spermidine induces autophagy and prolongs the lifespan of aging animals (LaRocca et al., 2013).

## Anti-aging interventions: Clinical studies lack validated data

In humans, there is a lack of validated information for most anti-aging medications (Liu, 2022). While multiple studies show the potential positive effects of different compounds in various living organisms, most lack clinical evidence in humans. Many of the interventions balance between expected positive effects and unavoidable adverse side effects.

Metformin reduced levels of IL-6, TNF-α pro-inflammatory cytokines in the serum, and MCP-1 in the urine of T2D patients and reduced the inflammatory responses in the systemic circulation (Cameron et al., 2016). Several studies showed that metformin is effective against cognitive decline, while others claimed that exposure to metformin contributes to neurodegeneration, Parkinson and Alzheimer’s disease (Ng et al., 2014; Hervas et al., 2017). DM2 patients treated with metformin as monotherapy had better survival than non-diabetic matched controls (Bannister et al., 2014).

Rapamycin, combined with medications capable of controlling the adverse effects of rapalogs, such as statins and metformin, might be a promising strategy to control age-associated diseases (Martinet et al., 2014; Blagosklonny, 2017; Leontieva and Blagosklonny, 2017). Rapamycin analogs, everolimus, and temsirolimus may delay age-related processes, including neurodegenerative disease and cardiac hypertrophy, and maintain stem cells’ regenerative capacity (Saraswat and Rizvi, 2017; Kraig et al., 2018).

Statins have anti-inflammatory, immunomodulatory, anti-oxidant, and antiproliferative effects, stabilize atherosclerotic plaques, prevent aggregation of platelets, and carry the potential for treatment and prevention of multiple age-related diseases (Kavalipati et al., 2015; Oesterle et al., 2017). Meta-analysis of 92 placebo-controlled and active-comparator trials demonstrated that statins are effective for primary and secondary cardiovascular prevention, reducing major coronary events and all-cause mortality by reducing oxidative stress and inflammatory markers (Naci et al., 2013; Shimomura et al., 2016; Gilbert et al., 2017). Statins induce apoptosis, cause cytostatic and antiproliferative effects, augment anticarcinogenic properties of standard chemotherapies, and attenuate cancer cell migration and invasion (Papanagnou et al., 2017).

Anti-lipolytic drugs mimic calorie restriction, triggering autophagy to alleviate the aging process. Anti-lipolytic drugs such as Acipimox reduce blood lipids, inhibit the release of free fatty acids from whole-body fat tissue, reduce the synthesis of cholesterol and triglyceride, reduce the total cholesterol level triacyl and low-density lipoprotein in plasma and exert anti-oxidant effects (Bao et al., 2020).

Regulation of inflammation can serve as an anti-aging intervention. However, the lack of large trials makes it difficult to draw final conclusions (Neves and Sousa-Victor, 2020). A meta-analysis of nine trials where aspirin was used for primary cardiovascular prevention in 100,076 patients showed that long-term aspirin reduced myocardial infarction, ischemic stroke, and all-cause mortality (Raju et al., 2016). A recent meta-analysis involving data from 36,196 patients did not confirm the protective effect of aspirin on cognitive decline in older age. Pooled data from RCTs and observational studies showed that low-dose aspirin was not associated with better cognition or improved cognitive impairment (Veronese et al., 2017).

Dietary supplementation with large doses of anti-oxidants had no preventive or therapeutic effect (Biswas, 2016; Ros and Carrascosa, 2020). A Mediterranean diet rich in monounsaturated fatty acids, such as olive oil, increases the telomerase length (Gomez-Delgado et al., 2018). Increasing the levels of beta-carotene and alpha-tocopherol in healthy adults’ diets protects telomere length (Yabuta et al., 2016). The Okinawan diet includes more fresh vegetables and fruits, and fewer proteins from fish and soy were proposed to improve health (Nilholm et al., 2018).

In humans, CR protects from arthritis, cardiovascular diseases, age-related diabetes, colon lesions, delayed osteoporosis, sarcopenia, brain atrophy, and cognitive decline, and decreases cancer incidence and progression (Balasubramanian et al., 2017). A low-calorie diet was associated with lower mortality, cardiovascular events, and cancer (Kagawa, 1978). Trials of CR in humans, the CALERIE trials (Comprehensive Assessment of Long-term Effects of Reducing Intake of Energy) and CRON (Caloric Restriction with Optimal Nutrition) showed that improved glucose tolerance, decreased LDL levels, decreased C-Reactive Protein levels, better blood pressure control, and reduced 10-year risk of cardiovascular events (wustlWUSoMCGWEPRSBVDTFLS-MKSKBKSHJOj et al., 2006; Larson-Meyer et al., 2010; Most et al., 2018). The CRON trial showed reduced risk factors associated with DM2 atherosclerosis and cardiovascular disease (Fontana et al., 2004; Fontana et al., 2010). CR decreases intramyocellular lipid content, blood pressure, total cholesterol, LDL cholesterol, C-reactive protein, TNFα, and 10-year risk of cardiovascular disease (Das et al., 2007; Rochon et al., 2011; Stewart et al., 2013).

Time-restricted feeding (TRF) limits the daily food intake to 8 h or less. Intermittent fasting (IF) designates eating patterns in which individuals go extended periods (e.g., 16–48 h) with little/no energy intake. The fasting and mimicking diets (FMD) include diets that mimic fasting, affecting the expression pattern of different tissues based on circadian signaling rhythm. TRF induces gene expressions, including mTOR and AMPK, that enhance the circadian clock’s oscillations and positively affect metabolic control. (Hatori et al., 2012) TRF without CR reduced metabolic alterations such as hyperinsulinemia, inflammation, hepatic steatosis, and insulin resistance (Hatori et al., 2012; Tinsley and La Bounty, 2015; Mitchell et al., 2019). FMD twice a month from middle age extends longevity, reduces visceral fat and cancer incidence, and rejuvenates the immune system while hampering bone mineral density loss (Brandhorst et al., 2015).

Numerous natural products may exert anti-aging properties, among them Arbutin, Ramulus mori extract, Licorice extract, Glabridin, Liquiritin, Kojic acid, Methyl gentisate, Aloesin, Azelaic acid, Vitamin C, Thioctic acid, Soya bean extracts, Niacinamide, α and β-hydroxy acids, Lactic acid, Chamomile extract, Ellagic acid, tea contains polyphenols, carotenoids, tocopherols, ascorbic acid, minerals, and certain phytochemicals (Zaid and Al Ramahi, 2019). A cross-sectional study found that using natural products by older adult Chinese men is positively associated with telomere length (Cabrera et al., 2006; Huang et al., 2016; Kamal et al., 2021).

Non-pharmacological approaches to delay age-related decline, including diet and physical activity, can provide some life-extending and disease-preventing benefits (McPhee et al., 2016; Di Daniele et al., 2017; Piskovatska et al., 2019). Hormesis postulates that mild to moderate biological stress can induce an adaptive response that activates several cellular pathways ameliorating lifespan and changing homeostasis. Oppositely, the stress of higher intensity can be deleterious (Gomez-Delgado et al., 2018).

The limited effectiveness of currently tested interventions on the potential to improve longevity and the complexity of developing new mechanisms and therapeutic targets implies a need to provide methods for enhancing the effectiveness of currently available regimens.

## Improving the response to chronic medications using the constrained disorder principle

Variability characterizes biological systems and provides a mechanism for surviving under numerous random internal and external stimuli (Ilan, 2019a; Ilan, 2019b; Ilan, 2019c; Ilan, 2019d; El-Haj et al., 2019; Ilan, 2019f; Ilan-Ber and Ilan, 2019; Ilan, 2020b; Forkosh et al., 2020). The constrained disorder principle defines aging as the malfunctioning of biological systems due to loss of variability or moving out of the boundaries of variability. The principle provides an anti-aging platform based on increasing the disorder in the natural system in cases where it is reduced or tightening disorder boundaries when the disorder is increased (Ilan, 2021b).

Anti-aging therapies mandate prolonged chronic treatment. One of the reasons for the lack of effectiveness of chronic pharmacological and non-pharmacological interventions is the human body’s compensatory responses, which lead to the development of tolerance to this intrusions (Ilan, 2020c). The compensatory mechanisms that lead to a lack of a sustainable response evolve from mechanisms that differ between subjects and interventions (Ilan, 2020c). The compensatory responses lead to partial or complete loss of response in up to half of the users of numerous agents, including anti-inflammatory agents, anti-cancer drugs; anti-depressants, anti-epileptics analgesics, medical cannabis-based products, metabolic agents, and others (Ilan, 2019e; Ilan, 2019f; Kenig and Ilan, 2019; Khoury and Ilan, 2019; Ilan, 2020c; Gelman et al., 2020; Ilan and Spigelman, 2020; Kessler et al., 2020; Potruch et al., 2020; Ilan, 2021a; Ishay et al., 2021a; Azmanov et al., 2021; Ishay et al., 2021b; Hurvitz et al., 2021; Isahy and Ilan, 2021; Kenig et al., 2021; Khoury and Ilan, 2021; Kolben et al., 2021; Azmanov et al., 2022; Gelman et al., 2022). Compensatory mechanisms also underlie the plateaus effects in training and other non-pharmacological interventions, hindering the ability to achieve a long-term effect (Gelman et al., 2022).

The “loss of complexity” mechanism accompanying the aging process suggests prescribing medications in an irregular, non-linear manner (i.e., unpredictable dosage change and drug administration timing) (Kyriazis, 2003). It has been proposed that other external stimuli, such as nutrition and social meetings, should also be planned to regain complexity (Kyriazis, 2003).

The constrained disorder principle provides a method for overcoming these compensatory effects using variability-based therapeutic regimens and anti-aging interventions (Ilan, 2021b). The principle provides a platform that can improve the effectiveness of current medications and non-pharmacological interventions. Regimens that showed promising results in pre-clinical models but failed to show similar results in humans and those with only limited results can be improved by implementing the constrained-disorder principle-based approach (Ilan, 2020d).

## The role of variability in anti-aging intervention

Several anti-aging regimens incorporate the concept of variability. While the mechanisms underlying metformin responses in humans remain largely unknown, they can be explained by genetic variability. Organic cation transporter 1 (OCT1) plays a role in the hepatic uptake of metformin and affects AMPK activation. In mice hepatocytes, deletion of OCT1 reduced the effects of metformin on AMPK phosphorylation and gluconeogenesis in mice. Subjects with OCT1 polymorphism have reduced responses to metformin (Soukas et al., 2019). In OCT1-deficient mice, the glucose-lowering effects of metformin were abolished. Seven polymorphisms of OCT1 that exhibited reduced metformin uptake were identified. Clinical trials have shown that glucose tolerance was significantly lower in individuals with reduced function polymorphisms. The data support that genetic variation in OCT1 may contribute to variation in metformin respones (Shu et al., 2007).

Metformin’s anti-aging effect varies between species (Soukas et al., 2019); studies have shown metformin to extend the longevity of certain organisms (worm, caged mice) but not others (fruit fly), a fact that may be attributed to their variable nutrient availability and their intensity of exercise. The glycemic effect of metformin varies between individuals, whereas researchers attempt to decipher the genetic and environmental differences distinguishing between responders to non-responders (Florez, 2017). The genetic variability in metformin metabolism is an example of reduced function polymorphisms and the differences between subjects.

The association between aging and gut microbiota constitutes an eminent illustration of variability’s application. Reduced gut microbiota diversity and increased colonization by opportunistic species characterize the process of aging. The gut microbiota of centenarians is more diverse than that of younger adults. (Cӑtoi et al., 2020) Age-related altered gut microbiota profile includes loss of genes for short-chain fatty acids (SCFA) production with decreased saccharolytic and increased proteolytic bacteria, leading to pathobionts’ dominance and contributing to inflamma-aging. While the described age-related alterations in the microbiome are between subjects and between older and younger individuals, intra-subject changes which evolve from responses to internal and external triggers are of importance (Ilan, 2019e).

A systematic review and meta-analysis determined the effect of short-term probiotic supplementation in older adults on the immune system. (Miller et al., 2019) A short-term, 3–12 weeks, probiotic supplementation improved the cellular immune function in healthy older adults. A longer duration of 6 months of probiotic supplementation altered the gut microbiota structure by inducing the proliferation of beneficial microorganisms and positively affecting individuals’ health. These data suggest that variability in the duration of administration impacts the outcome.

The data support that several anti-aging therapeutics are linked to the variability in their underlying mechanisms and molecular pathways. The heterogeneous responses to therapies can be attributed to genetic, metabolic, and environmental variability among subjects. While most studies focus on the heterogeneity between younger and aged populations and the inter-individual variability among the aged population, there is also an essential role for the intra-subject variability. Both inter and intra-subject variabilities are mandatory for proper flexibility and adaptability to continuous internal and external perturbations.

## The constrained disorder principle provides a method for improving the effectiveness of anti-aging therapies using second-generation artificial intelligence systems

The constrained disorder principle provides a platform for second-generation artificial intelligence (AI) systems that regulate therapeutic regimens and non-pharmacological interventions (Ilan, 2023). Second-generation AI systems are based on the concept that biological systems are dynamic, and their behavior continuously changes among and between subjects (Ilan, 2020d). These systems implement the n = 1 notion and closed-loops algorithms that constantly adapt to changes. They use but are independent of big data sets, which are the basis for most first-generation AI systems that lack accuracy and relevance to a single subject. Most big data sets prevent an ability of dynamicity, which is behind every biological function (Ilan, 2020c; Ilan, 2020d; Ilan, 2021b; Ilan, 2022).

Table 1 demonstrates examples of implementing the constrained disorder principle-based methods for improving anti-aging programs. Second-generation AI systems that implement variability within borders can be used with each example to improve the response to therapies and achieve a sustainable reaction.

**TABLE 1:** Implementing the constrained disorder principle-based methods for improving anti-aging programs.

Anti-aging program	The constrained disorder principle-based method
Pharmacological treatments	• Manipulating the dosage and intervals of anti-aging medications within approved ranges results in an irregular, complex, and non-stationary drug administration. Examples are metformin, rapamycin, statins, anti-inflammatory agents, and anti-oxidants
	• Changes in variability indexes (e.g., heart rate variability, blood pressure variability) can be used as input for the algorithm and as a tool to monitor medication response and direct further treatments
Diet and nutrition	• Daily alterations of eating habits include consuming various food products, calorie intake, and nutritional supplementations
	• Intermittent fasting
	• Implementing variability into dietary maneuvers
Physical exercise	• Modification of daily physical activity programs, intensity, timing, and duration for regaining physiological complexity
	• Wearable devices and accelerometers to track the variability in daily exercise patterns
Mental exercise	• Performing daily-changing unusual tasks that challenge various mental skills, such as puzzle solving, listening to or playing music, meditating, or using the non-dominant hand, may reduce cognitive impairment associated with aging
Social engagements	• Complex interactions with different people and social circles at different times may regain neuronal complexity and positively impact health
	• Introducing variability into these arrangements is expected to improve their effectiveness

The described second-generation AI systems provide a method for implementing intra-subject variability-based interventions generated based on capturing and quantifying inter and intra-individual variability signatures (Ilan, 2021b).

The second-generation AI algorithm is developed in three stages. The first is an open-loop system that sets up regimens comprising variability in the dosing and administration times of the intervention. This step is not dependent on input and does not account for the outputs. For aging, incorporating variability may be sufficient for improving clinically meaningful outcomes (Ilan, 2020d; Ilan, 2021b). The concept implies that for maximum benefit, anti-aging pharmacological and non-pharmacological interventions should be administered at irregular, pulsed, or multiple intervals and at changing dosage strengths. Maintaining various external and internal physiological stimuli, such as variable physical and mental exercise regimes, can slow the aging procesess (Kyriazis, 2003).

In the second stage, an outcome-based closed-loop system is implemented. The system adapts itself to clinically meaningful endpoints irrespective of the input. It provides a method for tailoring the degree of variability of the intervention in a personalized way. It can overcome the task in cases where the degree of disorder is challenging to measured (Ilan, 2020d; Ilan, 2021b).

In the third stage, the platform implements signatures of variability into the algorithm. This step is essential for improving aging processes, as relevant signatures may be lower or higher than required. An example is the use of heart-based variability regimens. HRV and HRF comprise measures of the intrinsic variability of cardiac function and correlate with cognitive functions. Irrespective of the method used for calculating the HRV, it is clear that the variability is altered with aging. Similarly, looking into inflamm-aging, where cytokine secretion variability is quantified and implemented as a dynamic variable into the algorithm’s input layer (Ilan, 2020d; Ilan, 2021b).

The digital pill comprises any drug that a second-generation AI-based app regulates its administration (Naci et al., 2013). The first level involves randomization of the dosing and times of administration of a drug within the approved ranges that the physician sets up. For the second level, clinical endpoints are pre-defined, and closed-loop tailors the variability to the treatment regimens in a personalized way. It enables tailoring the variability regiments to the patient’s response in a dynamic way that keeps changing. In the third level, the system quantifies the variability measures and implements them into the algorithm for generating therapeutic regimens to improve the response rate to chronic anti-aging therapies (Ilan, 2020c; Ilan, 2020d; Ilan, 2021b; Ilan, 2022). Quantifying measures of variability and implementing them into the algorithm in a patient-tailored dynamic way provide a method to incorporate personalized variabilities into treatment algorithms aiming at overcoming the loss of response (Ilan, 2021b).

**FIGURE 1:**
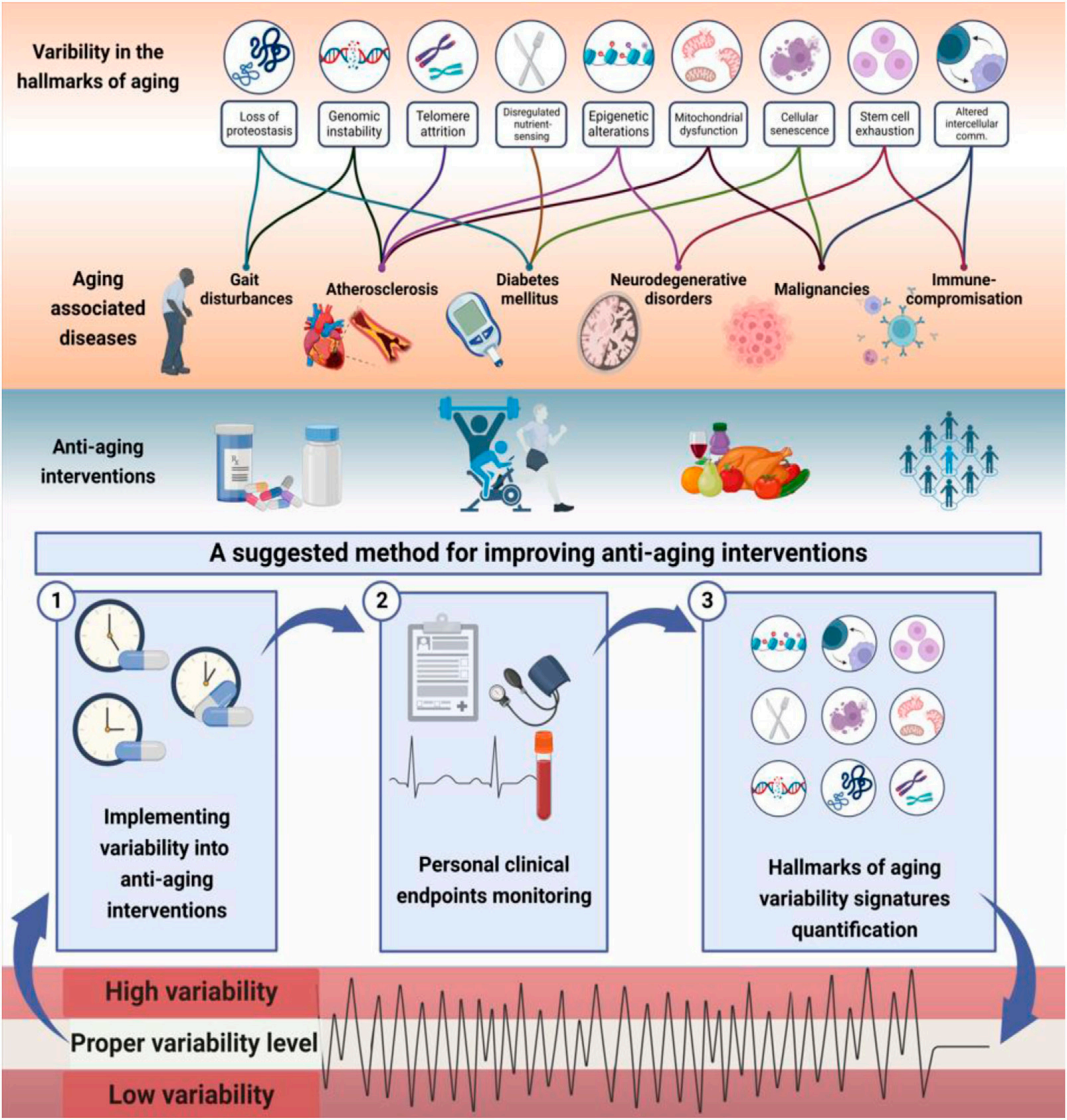
The hallmarks of aging are associated with altered variability and provide a basis for improving the effectiveness of anti-aging interventions: Illustrated is links between variability in hallmarks of aging and aging-related diseases. Higher or lower variability in molecular, intracellular, and intercellular mechanisms may result in accelerated aging. Per the constrained disorder principle, maintaining variability within a controlled range (in light green) may alleviate the aging process and improve anti-aging interventions. The system works in a three-stage process to implement variability into therapies within a determined range, track personal clinical endpoints, and dynamically modify the algorithm based on variability signatures. The potential of quantifying variability signatures of the hallmarks and implementing them into the algorithm is presented. The system enables enhancing internal variability when the degree of variability is too low or regulating the variability when it is too high (in red) to improve the effectiveness of anti-aging interventions.

Figure 1 presents a schematic description of the potential associations between the hallmarks of aging and the concepts of complexity and variability. It provides examples of links between the variabilities of aging hallmarks and age-related diseases. The figure illustrates the method for implementing variability into anti-aging interventions to improve their effectiveness using constrained-disorder principle-based second-generation AI systems. Per this principle, maintaining variability within a controlled range may alleviate the aging process and improve anti-aging interventions. The figure highlights the potential of quantifying variability signatures of the hallmarks and implementing them into the algorithm. It provides a comprehensive scheme associating the variability of the hallmarks with age-related diseases and personalized treatment algorithms, paving the way for improved targeted anti-aging interventions. The system enables enhancing internal variability when the degree of variability is too low or regulating it when it is too high.

Implementing variability into therapeutic regimens of anti-aging drugs, such as metformin, statins, anti-oxidants, and all types of medications and natural products, which showed some efficacy in pre-clinical and clinical studies, is expected to improve the response to these drugs. In parallel, using the digital pill with anti-depressants, anti-diabetics, cardiac drugs, chronic analgesics, anti-cancer drugs, and other medications which are commonly used in the aged population is expected to overcome the compensatory mechanisms and increase the response rate and the clinical benefits from these medications, improving quality of life and increasing lifespan (Ilan, 2019e; Ilan, 2019f; Kenig and Ilan, 2019; Khoury and Ilan, 2019; Gelman et al., 2020; Ilan and Spigelman, 2020; Kessler et al., 2020; Potruch et al., 2020; Ilan, 2021a; Ishay et al., 2021a; Azmanov et al., 2021; Ishay et al., 2021b; Hurvitz et al., 2021; Isahy and Ilan, 2021; Kenig et al., 2021; Khoury and Ilan, 2021; Kolben et al., 2021; Azmanov et al., 2022; Gelman et al., 2022).

The second-generation AI system can enhance the effectiveness of exercise, physiotherapy, and mind-targeted regimens, by implementing controlled variability into the interventions (Gelman et al., 2022). Using the constrained-disorder principle-based, AI is expected to improve the response to these interventions and ensure a long-term sustainable response (Gelman et al., 2022). Weight regain is a significant obstacle to the use of anti-aging dietary regimens. The compensatory mechanisms that evolve from altered hormone secretion and chronobiology mechanisms imply a lack of sustainable effects of the dietary-based intervention (Khoury and Ilan, 2021). Using a second-generation AI system provides a method for ensuring the long-term effectiveness of dietary procedures by introducing variability into the diets, thus overcoming the compensatory metabolic responses which hinder long-term responses (Khoury and Ilan, 2021).

## Summary

Aging is a multifactorial process, and it is unlikely that a single mechanism, and thus a single agent, can resolve it. Altered variability and complexity are found in aging and may underlie some of the above-described tools. Studies showed decreased or increased variability in older adults, per the constrained disorder principle, which implies getting the variability out of the dynamic boundaries. While multiple attempts to improve longevity with numerous compounds are continuously being tested pre-clinically and clinically, their overall effectiveness is limited and lacks validation. The described second-generation AI-based platform provides a system for improving the effectiveness of the currently used drugs and interventions. Ongoing studies are expected to shed light on their potential for overcoming the lack of response to interventions and increasing longevity.

## Abbreviations

AI: artificial intelligence; DNA: deoxyribonucleic acid; miRNA: micro-ribonucleic acid; ADL: activities of daily living; HRV: heart rate variability; ECG: electrocariogram; HRF: heart rate fragmentation; EEG: electroencephalogram; BPV: Blood pressure variability; fMRI: functional magnetic resonance imaging; NF-κB: nuclear factor kappa-light-chain-enhancer of activated B cells; Wnt: wingless related integration site; TERT: telomerase reverse transcriptase; SIRT/SIR: sirtuin/silent information regulator; IGF: insulin/insulin-like growth factor; mTOR: mamlian target of rapamycin; SASP: senescence-associated secretory phenotype; RNA: ribonucleic acid; IL: interleukin; VEGF: vascular endothelial growth factor; TGF: transforming growth factor; JAK: Janus kinase; EV: extracellular vesicles; ROS: reactive oxygen species; NAD: nicotinamide adenine dinucleotide; CR: calorie restriction; PARP: polyadenosine diphosphate ribose polymerase; mDNA: mitochondrial deoxyribonucleic acid; ATP: adenosine triphosphate; TNF: tumor necrosis factor; AMPK: adenosine monophoshate-activated protein kinase; CRM: calorie restriction mimetics; 2DG: 2-deoxy-D-glucose; HO: heme oxygenase; AD: Alzheimer’s disease; GH: growth hormone; T2D: type 2 diabetes mellitus; AMP: adenosine monophosphate; mTORC: mamlian target of rapamycin complex; AGEs: advanced glycation end-products; COX: cyclooxygenase; HDAC: histone deacetylase; NRF: nuclear factor erythroid related factor; FOXO: forkhead box O; ARE: antioxidant responsive element; AQP:aquaporin; KD: ketonic diet; RCT: randomized controlled trial; TRF: time-restricted feeding; IF: intermittent fasting; FMD: fasting mimicking diet; OCT: organic cation transporter; SCFA: short chain fatty acids;.
